# Development of an In Vitro Method for Assessing the Potential Irritation of Medical Devices and OTC Products Used in the Oral Cavity

**DOI:** 10.3390/toxics13040233

**Published:** 2025-03-22

**Authors:** Christian Pellevoisin, Marek Puskar, Jennifer Molignano, Kaitlyn Coen, Mitchell Klausner, Silvia Letasiova

**Affiliations:** 1 MatTek Corporation, Ashland, MA 01721, USA; jmolignano@mattek.com (J.M.); kcoen@mattek.com (K.C.); mklausner@mattek.com (M.K.); 2 Urbilateria, 37540 Saint Cyr sur Loire, France; 3 MatTek Europe, 821 05 Bratislava, Slovakia; mpuskar@mattek.com (M.P.); sletasiova@mattek.com (S.L.)

**Keywords:** oral irritation, EpiOral^TM^, in vitro, OTC, medical devices

## Abstract

The aim of this study was to evaluate an in vitro method using the EpiOral^TM^ model, a three-dimensional cultured human buccal epithelium, for assessing the oral irritation potential of various products. We evaluated different concentrations of nine chemicals commonly found in over-the-counter (OTC) products and medical devices, including chlorhexidine digluconate, sodium hypochlorite, phosphoric acid, hydrogen peroxide, lactic acid, ethanol, sodium dodecyl sulfate, 1-decanol and methyl methacrylate. The method was able to identify the irritants with a clear dose–response relationship between cell viability and an increasing concentration of the chemicals in the tested solutions. Using three exposure times (1, 4 and 18 h) and calculating the ET-50 (time required to induce a 50% reduction in cell viability), the solutions were classified according to their irritant potency (strong, moderate, mild or non-irritant). The results showed excellent correlation with historical in vivo data by matching the potency classifications in most cases. This study highlighted the importance of multiple exposure times for accurate assessment, as some solutions with irritant chemicals require longer exposure to produce effects. By providing information on both the irritant potential and potency, this method proved useful for toxicologists in the risk assessment of OTC products and medical devices that come into contact with the oral cavity.

## 1. Introduction

The oral cavity is contacted by different types of products for a variety of uses related to hygiene, dental care, and orthodontic treatment. The products can be broadly categorized into over-the-counter (OTC) products, cosmetics, and medical devices. In the United States, OTC products are regulated by the Food and Drug Administration (FDA) under the Federal Food, Drug, and Cosmetic Act (FD&C Act). OTC products are those that can be purchased without a prescription and are used for personal care, hygiene, or minor health issues (e.g., mouthwashes, toothpastes, whitening strips, denture adhesives, etc.). In comparison, in the European Union, these products are generally regulated under the Cosmetics Regulation (EC) No 1223/2009 for cosmetic products or other relevant directives, depending on the product type. Medical devices have a medical purpose and cover dental implants, orthodontic devices (e.g., braces, aligners), dental cements, fillings and adhesives. They are regulated in the United States by the FDA under the Medical Device Amendments to the FD&C Act. In the European Union, the notified bodies are responsible for ensuring the conformity of medical devices with the Medical Device Regulation (MDR, Regulation (EU) 2017/745).

Whatever the category of these products, their local tolerance and that of their ingredients must be systematically evaluated. Oral irritation is an adverse effect that may be induced by contact of the mucosal oral cavity with these products. Some common symptoms of oral irritation include redness, swelling, itching, burning and soreness, and in severe cases, it can lead to difficulty eating, speaking or swallowing. To evaluate oral irritation, the animal is still the preferred model. For medical devices, the ISO 10993-23 standard [1] requires oral irritation testing to be performed on Syrian hamsters. Although this standard requires in vitro testing on a reconstituted human epidermis (RhE) before considering in vivo testing, this is not applicable to medical devices that come into contact with mucosal tissues. In this case, it is recommended to consider the use of other in vitro models with relevant cells or tissues if they are qualified for use with medical devices. To achieve this aim, different 3D models of human oral epithelia reconstructed from primary oral cells (e.g., EpiOral^TM^ and EpiGingival^TM^, MatTek Corporation and MatTek Europe) or immortalized cell lines (i.e., SkinEthic^TM^ HOE, Episkin) [2] are available commercially. These models are manufactured under highly controlled conditions and meet the requirements of quality standards for high levels of reproducibility. They have been used for years to model the oral cavity for different applications, including studying in vitro permeation of drugs [3,4], biofilm formation, bacterial and fungal colonization [5], tobacco products [6], and oral care product irritation [7,8,9]. Nevertheless, as far as local tolerance is concerned, these methods are only used for screening purposes, and none of them are qualified to replace the in vivo tests that are required under certain regulations.

The objective of this work was to assess the performance of an in vitro method to replace the animal test for oral irritation using a reconstructed oral epithelium model. Since 60% of the oral cavity is covered by non-keratinized mucosal lining epithelium [10], we chose to use the EpiOral^TM^ model. This model is made of normal, human-derived oral epithelial cells cultured to form a multilayered and highly differentiated 3D model of the human buccal phenotype. The absence of a keratinized layer in this 3D mucosal epithelium gives the method greater sensitivity than a 3D gingival (highly keratinized) epithelium model. EpiOral^TM^ is produced in accordance with good manufacturing procedures (GMPs) in the USA, Europe and China, making it widely available to the industry. We assessed the performance of the in vitro method compared to historical in vivo data by testing solutions with different compounds used in OTC products or medical devices: sodium dodecyl sulfate, chlorhexidine digluconate, hydrogen peroxide and ethanol. Given the limited in vivo data available, we extended our in vitro data to compounds often used in oral care products, such as lactic acid, methyl methacrylate, phosphoric acid, sodium hypochlorite, 1-decanol. Our results showed the excellent in vitro/in vivo correlation and capacity of the method to identify a wide range of reaction intensities, making it suitable for different contexts of use.

## 2. Materials and Methods

### 2.1. Tissue Preparation

**Figure 1:**
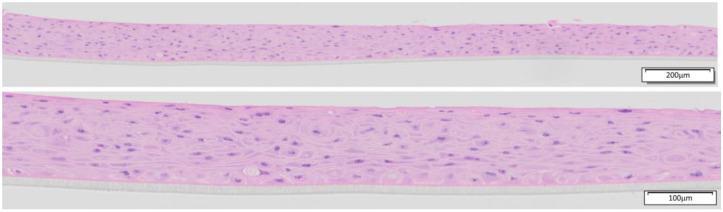
H&E-stained histological cross-sections of EpiOral tissue produced in 2023 by MatTek Europe at low (10× objective) and higher (20× objective) magnification.

The EpiOral tissues (ORL-200) were produced by MatTek Corporation (Ashland, MA, USA) and MatTek Europe (Bratislava, Slovakia). The EpiOral tissues (ORL-200) consist of normal, human-derived oral epithelial cells. The cells have been cultured on specially prepared cell culture inserts (0.6 cm^2^ surface) in serum-free medium to form highly differentiated 3D models of the human buccal phenotype. The EpiOral tissue model exhibits non-cornified, in vivo-like morphological characteristics, which are uniform and highly reproducible (Figure 1).

### 2.2. Quality Control of the Tissues

EpiOral (ORL-200) tissue is manufactured in accordance with good manufacturing procedures (GMPs) and each lot is evaluated prior to shipping. Each tissue lot must meet the quality control (QC) criteria that were first established in 2014. The purpose of the QC assay is to ensure reproducible tissue properties across independent tissue lots produced over time—an essential property for any toxicological test system [11]. The QC acceptance criteria were established based on QC data from 46 tissue lots produced during 2012–2013, following storage under conditions that simulate normal shipping to testing labs and commercial customers. EpiOral QC testing utilizes the ET-50 and the optical density (OD) from the MTT assay for tissue exposed to the negative control (NC), ultrapure water (H_2_O). The ET-50 refers to the exposure time required for the reference chemical, 1.0% (*v*/*v*) Triton X-100, to reduce the tissue viability to 50%, as measured by the MTT assay [7]. The intra-lot reproducibility of the EpiOral tissue is assessed by calculating an average coefficient of variation (CV) for the QC assay. Each lot’s ET-50 is determined by mathematically interpolating between the tissue viabilities for 3 different exposure times. The CVs for the viability at the three exposure times and for the negative control tissues are averaged to obtain an average CV% for the tissue lot.

### 2.3. In Vitro Irritation Assay

Following overnight storage at 2–8 °C (to mimic standard delivery times), the EpiOral tissues were pre-incubated for 1 h in 6-well plates with 0.9 mL of fresh medium under standard culture conditions (5% CO_2_, 37 °C). The solutions to be evaluated were prepared on the day of the experiment by dilution of the chemicals at the selected concentrations in polar (saline, 0.9% NaCl) or in non-polar solvent (sesame oil, SO) for chlorhexidine digluconate. Following the 1 h pre-incubation, the tissues were transferred to 0.9 mL of fresh medium and 100 μL of the test solution was applied to the apical surface of N = 2 tissues. The negative controls (NCs) for each time point were treated with Dulbecco’s phosphate-buffered saline (DPBS) and the positive controls (PCs) were treated with a 1% sodium dodecyl sulfate (SDS) solution in the corresponding vehicle (NaCl or SO). The exposure was stopped by washing the tissues with DPBS after 1, 4, or 18 h. The cell viability of the tissues was determined using the MTT viability assay. A list of the test chemicals and their in vivo GHS skin irritation category is given in Table 1.

### 2.4. MTT Viability Assay

Following treatment with the various test articles, the tissue viability was determined using the MTT viability assay. After exposure to the test articles and washing with DPBS, the tissue inserts were transferred to 24-well plates containing 300 μL of 3-(4,5-di-methylthiazol-2-yl)-2,5-diphenyltetrazolium bromide (MTT) solution (1 mg/mL, MatTek) and incubated at 37 °C, 5% CO_2_ for 3 h. After 3 h, the tissue inserts were transferred to new 24-well plates containing 2.0 mL of 2-propanol. The inserts were submerged in the 2-propanol, the 24-well plates were sealed in plastic bags, and formazan extraction was allowed to proceed overnight at room temperature in the dark. Following overnight extraction, 200 μL aliquots of the extractant from each control and test sample were dispensed into 96-well plates with wells filled with 200 µL of 2-propanol to be used as a blank subtraction for the viability calculation. The optical density (OD) absorbance of the extractant solution was measured at 570 nm using a plate reader and the tissue viability was calculated using the following equation:

$$\%\;viability=\left(\frac{OD\;treated\;tissue-OD\;blank}{OD\;control\;tissue-OD\;blank}\right)\times 100$$

### 2.5. ET-50 Calculation

Using a semi-log scale, the % viability (linear y axis) was plotted versus the dosing time (log x axis). For a given concentration, an ET-50 was interpolated using the exposure times for which the viability straddled 50%. Semi-log linear interpolation was performed with the equation Viability = m × log(time) + b, where m and b are coefficients to be determined based on the (viability, time) data points. Once m and b are determined, the viability is set to 50% and the equation is solved for the time, which is the ET-50. If a 50% decrease was reached within the first hour, the ET-50 was scored < 1 h and the test article was considered highly irritating. When the calculated ET-50 was >1 h and <4 h, the product was considered a moderate irritant and when the ET-50 was >4 h and <18 h, the product was considered a mild irritant. If the ET-50 was > 18 h, the material was considered non-irritating (Table 2).

## 3. Results

### 3.1. Quality Control of EpiOral Tissue

Table 3 shows the QC data for the EpiOral tissue produced during the periods 2016–2024 (MatTek Corporation) and 2022–2024 (MatTek Europe). The QC specifications for acceptable batches of ORL-200 are as follows: 34.85 < ET-50 < 105.78 min. In addition, the OD for the NC controls must be ≥ 1.00. As shown, the ET-50 values for the EpiOral tissues produced at both facilities meet the acceptance criteria first established based on QC data from 46 tissue lots produced during 2012–2013. Thus, the EpiOral tissue properties, in particular, the barrier properties, have remained constant since the QC specifications were established. The CV%, which is a good measure of the intra-lot reproducibility of the tissues, has been consistently below 10%. This verifies the high intra-lot reproducibility of the EpiOral tissue.

### 3.2. Effect of Concentration and Exposure Time

Although all nine chemicals tested are classified as skin irritants or corrosives (Table 1), the risk of oral irritation only arises at concentrations exceeding a specific threshold concentration for each compound. A dose–response relationship was observed for all the chemicals, as reflected by the decreased tissue viability as the chemical concentration increased (Table 4). This effect was observed at all three exposure times (1, 4 and 18 h) for all the test articles except for methyl methacrylate, which required at least 4 h to observe significant reduction in tissue viability, and for 1-decanol, which required 18 h to observe a dose–response effect. Note: During the preparation of the chlorhexidine digluconate solutions in saline (0.9% NaCl), a white precipitate formed. This phenomenon, known as the salting-out effect, occurs because chlorhexidine molecules react with chloride ions from NaCl to form chlorhexidine dihydrochloride, which is poorly soluble in water [13]. To remedy this situation, a non-polar vehicle referenced in ISO 10993-23, sesame oil, was used instead of saline to prepare the different concentrations of chlorhexidine.

### 3.3. ET-50 and Irritant Potency

To compare the relative toxicities and to enable categorization of the tested solutions, the ET-50s for the different concentrations tested were calculated (Table 5). If the tissue viability was below 50% after 1 h exposure, the ET-50 could not be calculated and the solution was classified as a strong irritant, possibly corrosive. Conversely, if the tissue viability was more than 50% after the 18 h exposure, an ET-50 could not be calculated and the solution was classified as non-irritant. When the tissue viability was 50% between 1 and 18 h, the ET-50 was used to determine if a solution was a mild or a moderate irritant (Table 2).

### 3.4. In Vivo/In Vitro Comparison

Table 6 shows the in vitro results of the EpiOral model and the results of work by Park et al. conducted in vivo on the Syrian hamster [14]. Based on the in vivo studies, the different test solutions were classified according to their degree of irritation: SDS (1%) > Triton X-100 (1%) ≈ hydrogen peroxide (3%) > ethanol (100%) ≈ chlorhexidine (0.2%, 2%). The in vitro results based on the ET-50 calculation not only made it possible to distinguish all the irritant solutions but also provided information on their potency, with a good correlation between the historical in vivo data in Syrian hamsters.

## 4. Discussion

Proper assessment of OTC products and medical devices helps ensure safe and effective use while minimizing the potential for adverse effects or complications. To replace the historical Syrian hamster cheek pouch test to assess oral irritation, there is a growing consensus on utilizing in vitro methods that use reconstructed buccal epithelium models made from human cells, such as the EpiOral^TM^ or the SkinEthic HOE models. Several articles have been published on these approaches, with the measured cell viability used as the main readout to predict the irritant potential, and some authors introduced cytokine release quantification (IL1α, IL8) as a secondary endpoint (Table 6). These tests are commonly employed to assess various oral hygiene products, cosmetics such as lipsticks, and specific chemicals identified as potential concerns found in OTC products and medical devices.

In our experiments, we tested different concentrations of nine chemicals commonly found in OTC products or medical devices: 1. chlorhexidine, an antimicrobial used in different oral care products, toothpastes or dental products; 2. sodium hypochlorite, widely used in dentistry at concentrations from 2 to 10% as an intra-canal irrigant to disinfect root canals [16]; 3. phosphoric acid, used in dentistry at a concentration between 30 and 40% for etching and cleaning tooth surfaces [16]; 4. hydrogen peroxide, found in concentrations ranging from 1% to 40% in dentistry products for tooth whitening, in antiseptic mouthwash or in cleaning agents; 5. lactic acid, a monomer of polylactic acid used in some materials and films in dentistry [17]; 6. ethanol, commonly used as a solvent and for its preservative and antiseptic properties in oral care products, used at a range from 5 to 26%; 7. SDS, a common ingredient in oral care products like toothpastes at concentrations of 0.5–2% [18]; 8. methyl methacrylate (MMA), a monomer of acrylic resin, with a wide variety of applications in dentistry for the fabrication of dentures, artificial teeth, and other dental prostheses [19]; and 9. 1-decanol, which despite not being used in oral care products, is an example of a mild irritant (GHS category 3) similar in structure to longer-chain alcohols, such as cetyl alcohol, stearyl alcohol, and cetearyl alcohol, that act as an emollient in oral care products [20].

We used a volume of 100 microliters (166 µL/cm^2^) for tissue dosing, which is considered suitable for maximizing sensitivity when testing mixtures rather than neat chemicals with 3D air–liquid interface models [21]. The results showed the sensitivity of the EpiOral model, with a clear dose–response effect for all nine tested chemicals. The experimental protocol provided data on the kinetics of the tissue viability vis-à-vis the exposure time and enabled the calculation of the ET-50, the time required for a 50% reduction in tissue viability. When a 50% viability reduction is reached before 1 h exposure, the ET-50 cannot be calculated and the solution is classified as a strong irritant. In this situation, and if necessary to de-risk a product with, for example, a very short contact time with the human oral mucosa, it may be possible to apply exposure times of less than 1 h in an attempt to calculate the ET-50.

Using this ET-50 approach, it was possible to not only assess the potential irritancy of the different tested solutions (strong, moderate or mild) but also to compare the current in vitro results with those of the 2015 publication of Park et al. [14]. In this publication, six products were tested in vivo according to ISO 10993-10:2010 (since 2021, oral irritation testing is integrated into the ISO 10993-23) to evaluate the optimum conditions for oral mucosal irritation testing using the hamster cheek pouch model. Of the six products, only Triton X-100 1% was not tested in our experiments. However, ET-50 Triton X-100 1% is used for the quality control of all batches of EpiOral and hence we added it to our in vitro/in vivo comparison above (Table 6). The six solutions classified as irritants in vivo were also classified as irritants with the in vitro method. Moreover, of the six products, four were given the same potency. Chlorhexidine 2% was over-classified as strong irritant in vitro rather than mild in vivo, and hydrogen peroxide 3% was classified as mild rather than moderate. The significance of the observed difference should be modulated in light of the fact that we only have one in vivo study available and that great variability in the subcategorization has been described in other in vivo irritation tests for which numerous historical data are available [22,23]. The information on potency might be important from the point of view of assessing the risk of mixtures compared with a method whose aim is to identify the intrinsic hazard of a pure chemical product. Previous studies have demonstrated the applicability of the EpiOral model for the evaluation of mixtures and commercial formulations [7,8,9,24]. However, as the products tested are often non- or low irritants, it would be instructive to apply the spiking approach presented in this study to complex mixtures and formulations.

The current multi-exposure time protocol has an advantage over single-exposure time protocols (Table 7). In some cases, the use of a single exposure time without information on the kinetics of toxicity will lead to an underestimation of the irritation risk of a solution compared to in vivo tests. For example, the 0.2% solution of chlorhexidine was determined to have an ET-50 of 12.6 h and therefore was categorized as a mild irritant (Table 2), which matches the in vivo result (Table 6). However, the use of a single exposure time with an exposure time < 12.6 h would incorrectly classify this solution as a non-irritant. Similarly, the 3% hydrogen peroxide solution was identified as a moderate irritant in the in vivo studies (Table 6). However, Yang et al. [8] used an exposure time of 90 min and classified the solution as a non-irritant on the basis of the tissue viability. Only the use of a second readout, quantification of IL-1α or histology, made it possible to identify the irritant potential of this solution. Using the current protocol, an ET-50 of 4.7 h was obtained and the solution was classified as a mild irritant, with an ET-50 slightly above the moderate irritant cutoff of ET-50 of 4.0 h. Notably, the dental literature reports several cases of chemical burns resulting from 3% hydrogen peroxide solution misuse [25,26].

## 5. Conclusions

The EpiOral tissue model has exhibited stable properties since the QC parameters were established for the tissue more than 10 years ago. Thus, the model is suitable for on-going evaluation of oral care products and biomedical devices contacting the oral cavity for regulatory purposes. The in vitro method using the EpiOral model of human oral epithelium allowed us to identify the irritant potential of the nine products tested at different concentrations, with an excellent correlation to the historical in vivo data. One of the strengths of this method is its capacity to provide more than just a binary yes/no answer regarding irritancy. By using three different exposure times, the test can determine the potency of irritant products. This nuanced approach allows for a more detailed understanding of mixtures and finished products affecting the oral mucosa. Given the heterogeneity of the products to be tested (dentifrices, mouth washes, OTC oral care products, and medical devices) and usage scenarios (duration of contact and frequency of use), information on the potency of the solution makes it an invaluable tool for toxicologists in their risk assessment processes. The next step will be to bring the subject to the level of working group 8 of ISO technical committee 194, in charge of the ISO 10993-23 standard for irritation of medical devices. The objective would be to work with the group’s experts to identify any additional data that may be needed and to consider appropriate steps for incorporating this type of in vitro method for oral irritation into ISO 10993-23.

## Figures and Tables

**Table 1:** List of the 9 chemicals and 2 solvents selected based on their use in OTC products or medical devices used in the oral cavity. On the day of the experiment, the chemicals were diluted to different concentrations in saline or sesame oil (SO).

Chemical	CAS No.	Initial Conc	Skin Irritation (GHS)	Manufacturer
Sesame oil (SO)	8008-74-0		NC	Merck, Darmstadt, Germany
Saline (0.9% NaCl)	7647-14-5	≥99%	NC	Merck, Darmstadt, Germany
Lactic acid	50-21-5	90%	2	Merck, Darmstadt, Germany
Sodium dodecyl sulfate (SDS)	151-21-3	20%	2	Merck, Darmstadt, Germany
Methyl methacrylate	80-62-6	100%	2	Merck, Darmstadt, Germany
1-decanol	112-30-1	98%	3	Merck, Darmstadt, Germany
Ethanol	64-17-5	100%	2	Merck, Darmstadt, Germany
Chlorhexidine digluconate	18472-51-0	20%	2	Merck, Darmstadt, Germany
Hydrogen peroxide	7722-84-1	30%	2	Merck, Darmstadt, Germany
Sodium hypochlorite	7681-52-9	6–14%	1	Merck, Darmstadt, Germany
Phosphoric acid	7664-38-2	≥85%	1	Merck, Darmstadt, Germany

**Table 2:** Potency classification from the calculated ET-50 values. This classification is adapted from the MTT Effective Time 50 (ET-50) for use with the EpiDerm Skin Model (EPI-200) [12].

ET-50 (Hours)	Classification
≤1	Strong irritant/possibly corrosive
1–4	Moderate irritant
4–18	Mild irritant
≥18	Non-irritant

**Table 3:** Results of the quality control (QC) testing—results for standardized QC testing for EpiOral tissue lots produced by MatTek Corporate (US) and MatTek Europe (Slovakia) after packaging and overnight storage at 4 °C. The ET-50 for the positive control (PC), 1% Triton X-100, and the optical density (OD) from the MTT assay for tissue exposed to the negative control (NC), ultrapure water (H_2_O), are shown. The coefficient of variation (CV%) is the average coefficient of variation for the 3 PC exposure time points and the NC tissues. The QC criteria listed were established based on data from 46 tissues lots produced during 2012–2013, following packaging and overnight storage at 4 °C.

	MatTek Corporation				MatTek Europe (Start of Production in 2022)			
Year	ET-50 (min)	H_2_O (OD)	CV (%)	Tissue Lots	ET-50 (min)	H_2_O (OD)	CV (%)	Tissue Lots
2024	48.5	1.507	9.1	23	64.4	1.731	7.9	31
2023	78.8	1.548	7.5	30	80.5	1.750	6.5	20
2022	86.7	1.503	7.8	30	70.1	1.689	7.5	14
2021	56.0	1.570	6.9	24				
2020	58.6	1.561	6.4	24				
2019	67.8	1.603	9.5	24				
2018	78.3	1.559	8.5	27				
2017	88.4	1.535	8.7	23				
2016	82.8	1.615	7.4	24				

QC Criteria: PC: 34.8 min < ET-50 < 105.8 min. NC: OD > 1.0.

**Table 4:** Tissue viability (%) relative to the DPBS negative-control-treated tissues of the 9 chemicals tested at different concentrations in saline solution. (*) For chlorhexidine digluconate, the formation of a white precipitate when dissolved in saline solution led us to use a non-polar solvent, sesame oil (SO), as recommended in ISO 10993-23 for the irritation of medical devices.

Chemicals	Concentration	Exposure Time		
		1 h	4 h	18 h
Lactic acid	0.10%	93.1	90.2	101.2
	0.50%	88.2	81.1	81.6
	1%	90.7	38.8	18.7
	4%	11.1	9.9	9.0
	5%	14.7	8.9	12.3
SDS	0.10%	107.2	110.8	109.2
	1%	80.6	11.1	4.9
	3%	50.2	7.6	3.3
	5%	22.2	4.7	2.8
Methyl methacrylate	0.10%	103.1	92.4	109.5
	1%	95.1	90.8	100.3
	5%	102.4	101.3	94.1
	25%	108.9	104.7	91.6
	50%	108.9	8.2	22.6
1-decanol	1%	100.4	90.5	108.9
	5%	99.9	96.3	121.7
	10%	98.8	98.9	113.1
	100%	131.8	96.3	8.1
Ethanol	25%	84.2	101.9	87.9
	50%	71.1	70.0	39.1
	100%	55.6	23.9	7.6
Chlorhexidine digluconate *	0.2%	125.5	131.1	24.9
	1%	103.2	105.4	89.7
	2%	37.0	13.2	7.3
	10%	16.1	10.7	8.6
Hydrogen peroxide	1%	75.8	73.5	68.0
	3%	60.0	52.5	29.7
	10%	11.1	5.7	6.4
Sodium hypochlorite	0.1%	108.8	99.2	84.0
	0.2%	88.7	99.6	101.3
	1%	4.9	4.2	6.2
	2%	4.9	4.2	7.8
	10%	0.8	1.9	2.8
Phosphoric acid	0.1%	105.1	100.9	93.6
	1%	96.6	15.5	12.3
	5%	15.1	14.4	15.0
	10%	14.7	17.6	19.8
	25%	10.9	14.0	16.6
	50%	1.4	4.7	9.3

**Table 5:** ET-50 calculated from the tissue viability measured at 3 exposure times for different concentrations of the chemicals solubilized in saline or SO. “>18”: ET-50 cannot be calculated because cell viability is greater than 50% after 18 h of exposure; “<1”: ET-50 cannot be calculated because cell viability is less than 50% after 1 h of exposure. (-) concentration not tested.

Chemicals	Concentration (*w*/*v*)									
	0.10%	0.20%	1%	2%	3%	5%	10%	25%	50%	100%
Sodium hypochlorite	>18	>18	<1	<1	-	-	<1	-	-	-
Sodium dodecyl sulfate	>18		1.6	-	1.0	<1	-	-	-	-
Phosphoric acid	>18	-	2.2	-	-	<1	<1	<1	<1	-
Lactic acid	>18	-	3.0	-	-	<1	-	-	-	-
Hydrogen peroxide	-	-	>18	-	4.7	-	<1	-	-	-
Chlorhexidine digluconate	-	12.6	-	<1	-	-	<1	-	-	-
Methyl methacrylate	>18	-	>18	-	-	>18	-	>18	2.2	-
Ethanol	-	-	-	-	-	-	-	>18	10.6	1.3
1-decanol	-	-	>18	-	-	>18	>18	-	-	8.8

**Table 6:** Comparison of the classification of 6 products tested in the in vitro method with the EpiOral model and the in vivo test according to ISO 10993-10:2010 [15]. (I) Irritant—(*) ET-50 from the quality control of EpiOral models with 1% Triton X-100.

		In Vivo		In Vitro		
Test Solutions	Conc.	Park et al. [14]			ET-50 (Hours)	
SDS	1%	I	Moderate	I	1.8	Moderate
Triton X-100	1%	I	Moderate	I	0.5–1.8 *	Strong to moderate
Hydrogen peroxide	3%	I	Moderate	I	4.7	Mild
Ethanol	100%	I	Moderate	I	1.3	Moderate
Chlorhexidine	2%	I	Mild	I	<1	Strong
Chlorhexidine	0.2%	I	Mild	I	12.6	Mild

**Table 7:** Publication list of in vitro methods for oral irritation using reconstructed models of human epithelia.

Year	Model	Test Materials	Dosing	Exposure Times	Endpoint	Reference
2025	EpiOral	Oral care products	100 μL	4, 18 h	Cell viability, IL-1α	Pobis et al., 2025 [24]
2024	EpiOral	Y-4, RM-C, SDS	100 µL	0.33, 1, 6 h	Cell viability	Gutierrez et al., 2024 [27]
2023	EpiOral	Ingredients oral care and lipsticks, chemical eye irritants	100 µL	2 h	Cell viability	Aizawa et al., 2023 [9]
2021	EpiOral	Oral care, ethanol, SLS, H_2_O_2_	100 μL	1.5 h	Cell viability, IL-1α, IL-8	Yang et al., 2021 [8]
2016	EpiGingival	Oral care products		24 h	Cell viability	Moghaddam et al., 2016 [28]
2007	EpiOral	Oral care products	100 μL	1, 2, 4, 6, 18 h	Cell viability, ET-50	Klausner et al., 2007 [7]
2007	SkinEthic RHOE	Orthodontic wires	1 mm pieces	24 h	Cell viability	Vannet et al., 2007 [29]
2005	SkinEthic RHOE	Methylated b-cyclodextrin (RAMEB)	30 μL	1, 4, and 24 h	Cell viability, IL-1α, histology	Boulmederat et al., 2005 [30]

## Data Availability

The data that support the findings of this study are available from the corresponding author upon reasonable request.
